# VALIDITY OF FIELD TESTS TO ESTIMATE CARDIORESPIRATORY FITNESS IN CHILDREN AND ADOLESCENTS: A SYSTEMATIC REVIEW

**DOI:** 10.1590/1984-0462/;2017;35;2;00002

**Published:** 2017

**Authors:** Mariana Biagi Batista, Catiana Leila Possamai Romanzini, José Castro-Piñero, Enio Ricardo Vaz Ronque

**Affiliations:** a Grupo de Estudo e Pesquisa em Atividade Física e Exercício Universidade Estadual de Londrina (UEL), Londrina, PR, Brasil.; b Departamento de Educação Física, Escola de Educação, Universidade de Cádiz, Puerto Real, Espanha.; c Departamento de Educação Física. Grupo de Estudo e Pesquisa em Atividade Física e Exercício, UEL, Londrina, PR, Brasil.

**Keywords:** reliability, physical fitness, youth, systematic review

## Abstract

**Objective:**

To systematically review the literature to verify the validity of field-tests to evaluate cardiorespiratory fitness (CRF) in children and adolescents.

**Data sources:**

The electronic search was conducted in the databases: Medline (PubMed), SPORTDiscus, Scopus, Web of Science, in addition to the Latin American databases LILACS and SciELO. The search comprised the period from the inception of each database until February 2015, in English and Portuguese. All stages of the process were performed in accordance with the PRISMA flow diagram.

**Data synthesis:**

After confirming the inclusion criteria, eligibility, and quality of the studies, 43 studies were analyzed in full; 38 obtained through the searches in the electronic databases, and 5 through private libraries, and references from other articles. Of the total studies, only 13 were considered high quality according to the adopted criteria. The most commonly investigated test in the literature was the 20-meter shuttle run (SR-20 m), accounting for 23 studies, followed by tests of distances between 550 meters and 1 mile, in 9 studies, timed tests of 6, 9, and 12 minutes, also 9 studies, and finally bench protocols and new test proposals represented in 7 studies.

**Conclusions:**

The SR-20-m test seems to be the most appropriate to evaluate the CRF of young people with the equation of Barnett, recommended to estimate VO_2_ peak. As an alternative for evaluating CRF, the 1-mile test is indicated with the equation proposed by Cureton for estimating VO_2_ peak.

## RESUMO

**Objetivo:**

Revisar sistematicamente a literatura para verificar a validade dos testes de campo para avaliação da aptidão cardiorrespiratória (ACR) em crianças e adolescentes.

**Fontes de dados:**

Foram utilizadas as bases de dados: Medline (PUBMED), SPORTDiscus, Scopus, Web of Science, além das bases latino-americanas LILACS e SciELO. A busca compreendeu todo o período de existência das bases até fevereiro de 2015, em idioma inglês e português. Todas as etapas do processo foram previstas de acordo com o PRISMA.

**Síntese dos dados:**

Após verificação dos critérios de inclusão, elegibilidade e qualidade dos estudos, 43 trabalhos foram analisados na íntegra, sendo obtidos 38 por meio da busca nas bases de dados eletrônicas e cinco por meio de biblioteca particular e referências de outros artigos. Do total dos artigos, apenas 13 foram considerados de alta qualidade segundo os critérios adotados. O teste mais investigado na literatura foi o *shuttle run* de 20 metros (SR-20 m), contabilizando 23 trabalhos, seguido pelos testes de distâncias entre 550 metros e 1 milha com 9 estudos, os testes com tempos de 6, 9 e 12 minutos também com 9 estudos e, por fim, os protocolos de banco e novas propostas de teste representados por 7 trabalhos.

**Conclusões:**

O teste SR-20 m parece ser o mais apropriado para avaliação da ACR de jovens, com a equação de Barnett, recomendada para estimativa do VO_2_pico. Como segunda alternativa para avaliação da ACR, indica-se o teste de 1 milha e, a equação proposta por Cureton, para estimativa do VO_2_pico.

## INTRODUCTION

Physical fitness, in general, refers to a series of physical characteristics that are directly related to the ability of an individual to perform physical activity and/or exercise.^1^ In this sense, among its components, great emphasis has been given to cardiorespiratory fitness (CRF), also known as aerobic fitness or maximal aerobic power.^2^

CRF is currently considered an important marker of health in both adults^3^ ^,^ ^4^ and young people.^1^ ^,^ ^5^ Children and adolescents who present high values of cardiopulmonary indicators tend to present decreased risk factors for cardiovascular diseases such as obesity, high blood pressure, dyslipidemia, and insulin resistance, among others.^6^ In addition, prospective studies have indicated that high CRF during childhood and adolescence is associated with a healthy cardiovascular profile in adulthood.^7^

With regard to the assessment of CRF indicators, peak oxygen consumption (VO_2_ peak) is widely recognized as one of the best indices to measure aerobic power in young people.^2^ VO_2_ peak can be measured objectively and reliably in the laboratory, through direct analysis of the gases involved in pulmonary ventilation, while performing progressive and maximal tests on various ergometers. However, due to the high cost, use of sophisticated equipment, need for trained evaluators to administer the tests, and high time demand for each evaluation, its use becomes limited in environments such as schools, sports clubs, and population-based studies.^8^

Thus, application-based field tests, which provide the prediction of VO_2_ peak using mathematical models, are becoming an interesting alternative for the evaluation of CRF, since they demonstrate important advantages, such as low operating costs, ease of application and access to test locations, and the opportunity to evaluate a large number of subjects simultaneously.^9^ On the other hand, field tests for evaluating CRF use indirect methods to estimate VO_2_ peak and thus can present considerable measurement errors. Therefore, for a field test to be considered appropriate it should have good “validity”, i.e., produce good measures of the variable that it purports to measure. Thus, when choosing a field protocol from those proposed in the literature to evaluate CRF, it is important to check whether it is valid for the desired population.

Two decades after the first initiative which summarized the criteria related to the validity of the various field tests for the evaluation of physical fitness in young people, Castro-Piñero et al.^8^ proposed a more detailed and systematic way, taking into consideration the different levels of evidence for the validity of the various field tests, according to the established quality criteria for the studies analyzed. Thus, given the great speed in the production of current scientific literature showing new validity evidence, especially in childhood and adolescence, as this is an important phase for the detection of health hazards and the promotion of interventions for health issues, this type of study becomes necessary.

Given the above, the objective of this study was to systematically review the literature to verify the validity of field tests and to evaluate CRF in children and adolescents.

## METHOD

We systematically reviewed the literature using Medline (PUBMED), SPORTDiscus, Scopus, Web of Science, in addition to the Latin American databases, LILACS, and SciELO. The search comprised the period from the inception of each database until February 2015, in English and Portuguese. We opted to use only these two languages because the main studies were available in English, and we included Portuguese for our interest to provide this information.

The search strategy included the following keywords: validation studies, oxygen consumption, child, and adolescents. In the specific case of the Latin American databases, LILACS, and SciELO, similar key words were used because these databases have a limit for the search, and/or no records were found when we used many descriptors with Boolean operators.

The eligibility criteria of the articles were the main objective of the investigation being to test the validity of one or more field tests to estimate CRF and the investigated population being children and/or adolescents considered healthy, i.e., without any diagnosed condition or any problems that prevented the realization of motor tests and non-athletes.

**Figure 1:**
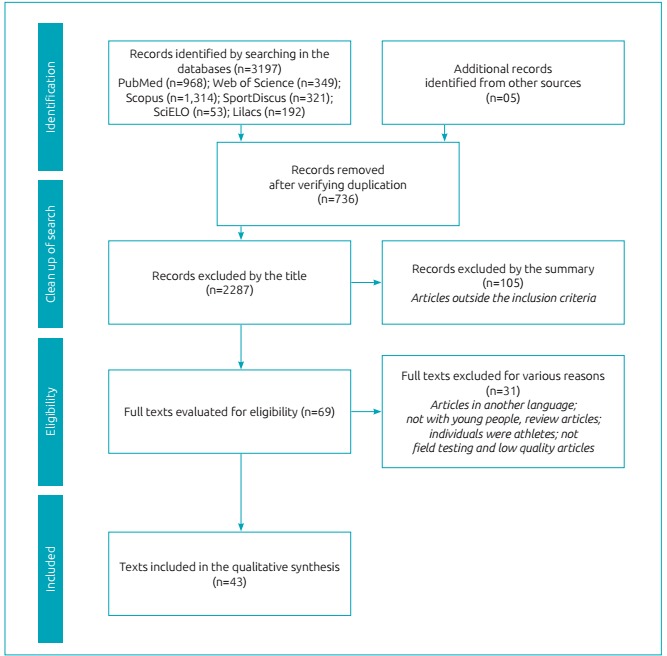
Flow Diagram of the article selection process.

All stages of the process were performed in accordance with the PRISMA flowdiagram^10^ (Figure 1) and the selection and analysis of the studies were conducted independently by two researchers (MBB and CLPR) and, in case of disagreement, a third researcher (ERVR) was invited to determine the inclusion or exclusion of studies.

After completion of the search, in accordance with the above procedures, 3,197 articles had been located in the six analyzed databases. Five additional studies were located and included by private libraries and bibliographic references. The next stage in the procedure was the exclusion of duplicate references and 736 references had been excluded, leaving 2,461 titles for analysis. The subsequent stage consisted of reading the titles of the papers selected for possible exclusion of those that did not meet the eligibility criteria; 2,287 papers were eliminated, leaving 174.

The abstracts were then read for more specific analysis of the criteria, for inclusion and exclusion of studies that had raised doubts during title analysis. 105 studies were excluded, as they did not meet the eligibility criteria, leaving 69 papers for the next stage. So, 25 other articles were excluded due to other reasons, leaving on the whole 44 papers.

The quality analysis was adapted from Castro-Piñero et al.^8^, and took into account three items in the studies: the number of subjects, the description of the sample, and the statistical analysis. In each of these items, the paper could receive a score between0 and 2 points, and at the end of the analysis, it was awarded a classification according to the sum of the points of each item. An adaptation of the score for the quality classification was made and categorized as: low (0-2 points), moderate (3-4 points) or high quality (5-6 points). After this evaluation, six articles were excluded due to low methodological quality.

It is worth mentioning that through analysis of the quality of the studies included in this systematic review, it was possible to establish levels of evidence as to the validity of the study protocols. As a standard strong evidence was attributed to testing protocols considered valid by 3 or more high-quality studies; moderate evidence was assigned to tests validated by 2 high-quality or 3 or more moderate quality studies, and limited evidence was attributed to tests validated by multiple low quality studies, inconsistent results of several studies independent of the quality, or the results of a single study

## RESULTS

The study selection process is exposed in Figure 1. The preliminary search yielded 3,197 articles, 968 in the Medline database (PUBMED), 349 in Web of Science, 1,314 in Scopus, 321 in Sport Discus, 53 in SciELO and 192 in Lilacs.

After analyzing the inclusion criteria and eligibility, a total of 3,153 studies were excluded up to this stage of the process. Further, the studies were classified according to the quality criteria. This procedure was adopted to ensure that only papers which had, at least a moderate methodological quality, were included, and therefore allowed at the end of the systematic review process levels of evidence, to validate the identification of the analyzed field tests.^8^ Furthermore, as Latin America databases were included in the search, we use the moderate evidence since some tests that are widely used in Brazil, for example 9 minute run/walk test, did not have any evidence validation.

Finally, 43 original articles were analyzed in full. Of the studies included in the review, 13 were considered high quality^11^ ^,^ ^12^ ^,^ ^13^ ^,^ ^14^ ^,^ ^15^ ^,^ ^16^ ^,^ ^17^ ^,^ ^18^ ^,^ ^19^ ^,^ ^20^ ^,^ ^21^ ^,^ ^22^ ^,^ ^23^ (Tables 1 and 2), and 30 as moderate quality (Tables 3 and 4). ^24^ ^,^ ^25^ ^,^ ^26^ ^,^ ^27^ ^,^ ^28^ ^,^ ^29^ ^,^ ^30^ ^,^ ^31^ ^,^ ^32^ ^,^ ^33^ ^,^ ^34^ ^,^ ^35^ ^,^ ^36^ ^,^ ^37^ ^,^ ^38^ ^,^ ^39^ ^,^ ^40^ ^,^ ^41^ ^,^ ^42^ ^,^ ^43^ ^,^ ^44^ ^,^ ^45^ ^,^ ^46^ ^,^ ^47^ ^,^ ^48^ ^,^ ^49^ ^,^ ^50^ ^,^ ^51^ ^,^ ^52^ ^,^ ^53^

**Table 1:**
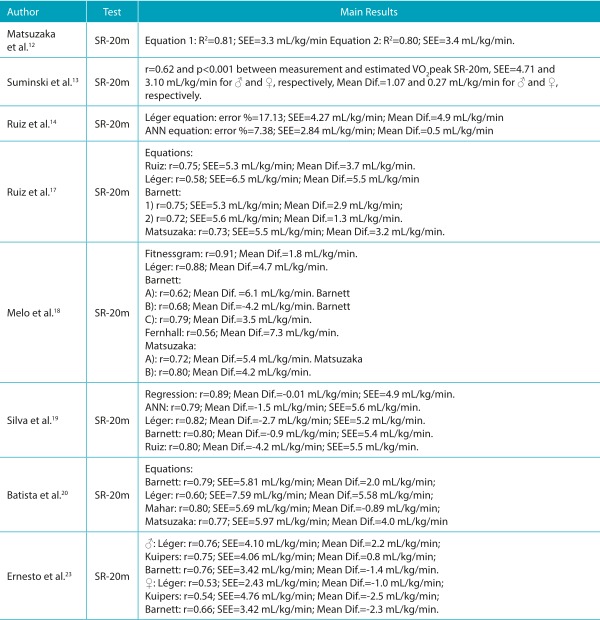
Summary of studies classified as high quality, aimed at validating SR-20m test for estimating cardiorespiratory fitness in children and adolescents.

♂ = boys; ♀ = girls; CRF: cardiorespiratory fitness; Mean Dif.: mean differences; VO_2_max or VO_2_peak: maximum oxygen consumption determined by the gold standard measure; r: correlation coefficient; R^2^: coefficient of explanation; SEE: standard error of estimate; mL/kg/min: relative values of oxygen consumption in milliliters per kilogram of body weight per minute; SR-20m: *shuttle run* test of 20 meters; ANN: mathematical model based on artificial neural network.

**Table 2:**
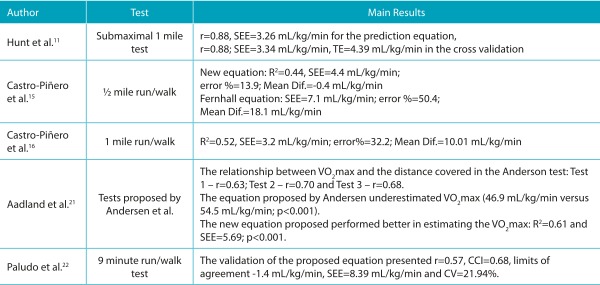
Summary of studies classified as high quality, aimed at validating other tests for estimating cardiorespiratory fitness in children and adolescents.

♂ = boys; ♀ = girls; CRF: cardiorespiratory fitness; Mean Dif.: mean differences; VO_2_ max or VO_2_ peak: maximum oxygen consumption determined by the gold standard measure; r: correlation coefficient; R^2^: coefficient of explanation; SEE: standard error of estimate; TE: total error; mL/kg/min: relative values of oxygen consumption in milliliters per kilogram of body weight per minute; CCI: confidence interval; CV: coefficient of variation.

**Table 3:**
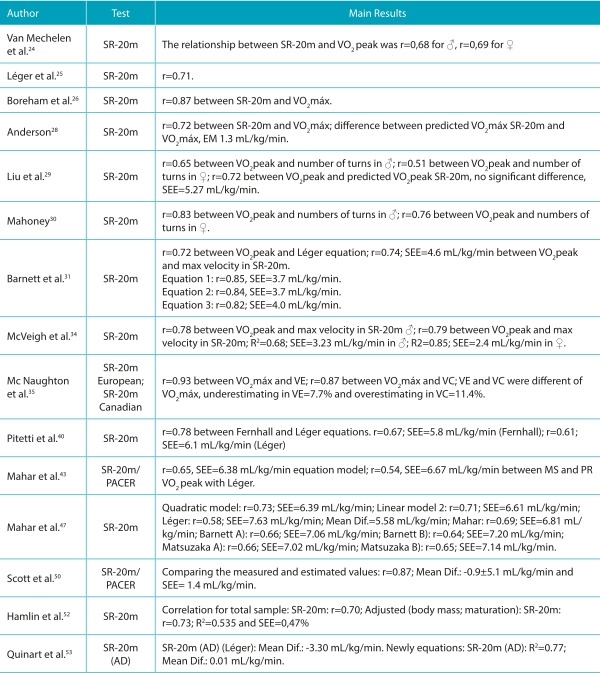
Summary of studies classified as moderate quality, aimed at validating SR-20m test for estimating cardiorespiratory fitness in children and adolescents.

♂ = boys; ♀ = girls; CRF: cardiorespiratory fitness; Mean Dif.: mean differences; MS and PR: Measured and Predicted; VO_2_max: maximum oxygen consumption; VO_2_ peak: peak oxygen consumption; CRF: cardiorespiratory fitness; SEE: standard error of estimate; %E: percentage of subjects who were within the measurement error; SR-20m: *shuttle run* test of 20 meters; PACER: Progressive Aerobic Cardiovascular Endurance Run; SR-20m (AD): *shuttle run* test of 20 meters adapted.

**Table 4:**
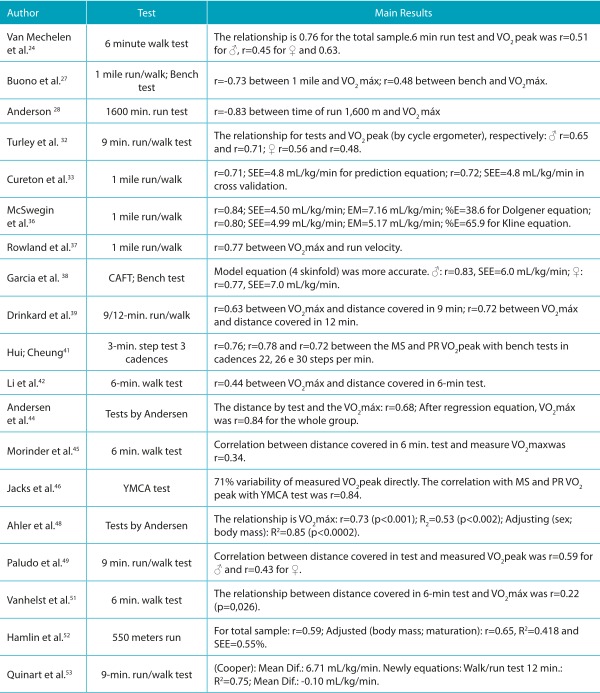
Summary of studies classified as moderate quality, aimed at validating other tests for estimating cardiorespiratory fitness in children and adolescents.

♂ = boys; ♀ = girls; CRF: cardiorespiratory fitness; Mean Dif.: mean differences; MS and PR: Measured and Predicted; VO_2_max: maximum oxygen consumption; VO_2_peak: peak oxygen consumption; SEE: standard error of estimate; %E: percentage of subjects who were within the measurement error; CAFT: Canadian Aerobic Fitness Test.

### 20 m Shuttle run test (SR-20 m)

Of a total of 23 studies that investigated the validity of the SR-20 m test, some sought to develop equations to estimate VO_2_peak^12^ ^,^ ^14^ ^,^ ^19^ ^,^ ^25^ ^,^ ^31^ ^,^ ^34^ ^,^ ^38^ ^,^ ^43^ ^,^ ^47^ ^,^ ^53^ including their regression models variables such as gender, age, speed obtained in the final stage of the test, number of turns, body weight, height, skinfold thickness, and body mass index (BMI), among others. The studies used linear mathematical and quadratic models and those based on artificial neural networks (ANN). Their results demonstrated correlation coefficient values between the VO_2_peak values from the new equation and those produced by the standard method ranging from r=0.65 to r=0.86, coefficients of determination between R^2^=0.68 and R^2^=0.85 and, standard error of estimates (SEE) from 2.4 to 7.0 mL/kg/min.

In addition, several studies carried out cross-validation of equations available in the literature, among which the most investigated was originally proposed by Léger et al. ^25^ , and analyzed in all the papers with this objective. ^13^ ^,^ ^17^ ^,^ ^18^ ^,^ ^19^ ^,^ ^20^ ^,^ ^23^ ^,^ ^28^ ^,^ ^29^ ^,^ ^35^ ^,^ ^40^ ^,^ ^47^ ^,^ ^53^

Other equations were also studied for validity, such as those created by Barnett et al., ^31^ ^,^ ^17^ ^,^ ^18^ ^,^ ^19^ ^,^ ^20^ ^,^ ^23^ ^,^ ^47^ ^,^ Fernhall et al., ^54^ ^,^ ^18^ ^,^ ^40^ ^,^ Ruiz et al., ^14^ ^,^ ^17^ ^,^ ^19^ ^,^ Matsuzaka et al., ^12^ ^,^ ^17^ ^,^ ^18^ ^,^ ^20^ ^,^ ^47^ , Mahar et al., ^43^ ^,^ ^20^ ^,^ ^47^ , and Kuipers et al. ^55^ ^,^ ^23^

However, in addition to determining the VO_2_ peak from the SR-20-m test, a simpler alternative and one widely used by professionals is the verification only of the parameters achieved in the test, such as the number of turns (back-and-forth) and the speed reached in the final stage of the test. In this sense, few studies had the objective of performing only the ratio of the VO_2_ peak evaluated in a direct manner in the laboratory, and the results in the SR-20-m test. ^24^ ^,^ ^26^ ^,^ ^28^ ^,^ ^29^ ^,^ ^30^ ^,^ ^35^ The results demonstrated correlation coefficient values ranging from r=0.51 to 0.93.

### Run and/or walk test over distances of 550 meters to 1 mile 

As a result of the systematic literature review, nine studies were found that investigated the validity of field tests to estimate CRF, with distances ranging from 550 meters running,^52^ 0.5-mile run/walk,^15^ 1-mile run/walk, ^16^ ^,^ ^27^ ^,^ ^28^ ^,^ ^33^ ^,^ ^37^ 1 mile walk,^36^ and 1-mile submaximal test.^11^ The results of these studies presented correlation coefficients ranging from r=0.59 to -0.83; coefficients of determination of R^2^=0.42 to 0.84, and standard error of estimates between SEE=3.26 mL/kg/min and 4.99 mL/kg/min.

In the case of the 1-mile run/walk test, some authors suggested equations for determining the VO_2_ max, such as, Buono et al.,^27^ , who obtained a coefficient of determination of R^2^=0.84 and standard error of estimate of 4.3 mL/kg/min or 9% for the proposed equation. Subsequently, Cureton et al.^33^ , proposed a generalized equation for individuals from 8 to 25 years of age, which considers information on total test time, age, sex, and BMI, and presented good validation values (r=0.72 and standard error of estimate of 4.8 mL/kg/min).

### Run/walk tests of 6, 9, and 12 minutes 

Nine of the studies analyzed investigated field protocols for evaluation of CRF with predetermined times, using a 6-walk, ^42^ ^,^ ^45^ ^,^ ^51^ 6-minute run, ^24^ 9-minute run/walk^22^ ^,^ ^32^ ^,^ ^39^ ^,^ ^49^ and 12-minute run/walk. ^39^ ^,^ ^53^

The tests with a time of 6 minutes involving walking and/or running presented results of the relationship between the distance obtained in the test and the VO_2_ peak evaluated in a direct method of between r=0.22 and 0.63, considered low to moderate, without proposing an equation to estimate the VO_2_ peak. ^24^ ^,^ ^42^ ^,^ ^45^ ^,^ ^51^ In the case of the test with a time of 9 minutes, the relationship between distance covered in the test and VO_2_peak was between r=0.43 and 0.71, ^32^ ^,^ ^39^ ^,^ ^49^ , with a proposed initiative for a prediction equation for VO_2_ peak using information on the distance covered in the test, biological maturation, sum of skinfolds, and sex, present in validation results of r=0.57, a mean difference of -1.4 mL/kg/min and SE=8.39 mL/kg/min.^22^

And finally, the tests which considered a time of 12 minutes running and/or walking, originally proposed by Cooper in 1968, presented correlation coefficients between the distance covered in the test, and VO_2_ peak evaluated in a laboratory of between r=0.70 and r=0.82,^39^ ^,^ ^53^ with a proposed equation to estimate VO_2_peak, but only in obese adolescents.^53^

### Maximal and submaximal bench tests or other protocols

Four studies were identified which sought to validate bench protocols, so as to estimate CRF. ^27^ ^,^ ^38^ ^,^ ^41^ ^,^ ^46^ In these studies, the tests had a duration of three minutes at different rhythms and paces. The relationship between the test results and the estimated VO_2_ peak measured directly, ranged from r=0.48 to 0.78. ^27^ ^,^ ^41^ Two studies proposed equations to estimate VO_2_ peak using the bench test, with results of r=0.77 to 0.84 and SEE between 6.0 and 7.0 mL/kg/min. ^38^ ^,^ ^46^

In addition, three papers were found dealing with a field test protocol to evaluate the CRF proposed by Andersen et al. ^44^ This test was initially proposed for young people aged 9 to 11 years, adolescent athletes between 14 and 15 years, and university students aged between 20 and 27 years. It has a duration of 10 minutes and is performed in a space delimited by two parallel lines, 20 meters from one another. The subject is required to run from one line to the other at intervals of 15 seconds running and 15 seconds resting, to complete the longest possible distance by the end of 10 minutes. The authors validated the test (r=0.68), and also proposed a prediction equation for VO_2_ peak which considered the maximum distance achieved in the test in meters, and sex (r=0.84).^44^

Subsequently, two studies have attempted to validate the test of Andersen et al.,^44^ as well as proposing a new prediction equation for VO_2_ peak, in other samples with ages ranging from 6 to 10 years. ^21^ ^,^ ^48^ . The validity results were considered satisfactory (r=0.63 to 0.73) and the proposed equation performed better than the original (R^2^=0.61 a R^2^=0.85; SEE=5.59 mL/kg/min).^21^

## DISCUSSION

After verifying the inclusion criteria, eligibility, and quality, 43 studies were analyzed in full. Of the total papers analyzed, 13 were considered of high quality and 30 were considered with moderate quality (Tables 1, 2, 3 and 4), according to the adopted criteria.^8^ The most commonly investigated test in the literature was the SR-20 m, accounting for 23 papers (Table 1 and 3), ^12^ ^,^ ^13^ ^,^ ^14^ ^,^ ^17^ ^,^ ^18^ ^,^ ^19^ ^,^ ^20^ ^,^ ^23^ ^,^ ^24^ ^,^ ^25^ ^,^ ^26^ ^,^ ^28^ ^,^ ^29^ ^,^ ^31^ ^,^ ^34^ ^,^ ^35^ ^,^ ^40^ ^,^ ^43^ ^,^ ^47^ ^,^ ^50^ ^,^ ^52^ ^,^ ^53^ which verified their validity and were included in the review, followed by tests of distances between 550 meters and 1 mile with 9 studies, ^11^ ^,^ ^15^ ^,^ ^16^ ^,^ ^27^ ^,^ ^28^ ^,^ ^33^ ^,^ ^36^ ^,^ ^37^ ^,^ ^52^ timed tests of 6, 9 and 12 minutes also with 9 studies^22^ ^,^ ^24^ ^,^ ^32^ ^,^ ^39^ ^,^ ^42^ ^,^ ^45^ ^,^ ^49^ ^,^ ^51^ ^,^ ^53^ and, finally, the bench and proposed new protocols which represented 7 papers (Tables 2 and 4). ^21^ ^,^ ^27^ ^,^ ^38^ ^,^ ^41^ ^,^ ^44^ ^,^ ^46^ ^,^ ^48^

### 20 m Shuttle run test (SR-20 m)

Our results corroborate those of Castro-Piñero et al.^8^ who found strong evidence that the SR-20 m is a valid test to estimate CRF in young people. However, with regard to the development of equations to estimate VO_2_ peak, some recently published papers complement these results regarding the cross validity of the equations available in the literature. ^18^ ^,^ ^19^ ^,^ ^20^ ^,^ ^23^ ^,^ ^47^ In the great majority of studies, the original equation proposed by Léger et al.^25^ presented results of lower validity, with a tendency to underestimate VO_2_ peak, compared to the models proposed later. However, when the analysis was stratified according to sex, the equation of Léger et al.^25^ produced better estimates of VO_2_peak for girls. ^18^ ^,^ ^20^ ^,^ ^23^

Prominent among the proposed equations were Barnett et al.^31^, with strong evidence of validity; Matsuzaka et al.^12^ with moderate evidence of validity; Ruiz et al.^14^ with moderate evidence of validity and Mahar et al. ^43^ ^,^ ^47^ also with moderate evidence of validity, despite being recently indicated by the FITNESSGRAM battery to estimate VO_2_ peak from the SR-20 m test. However, caution is necessary when interpreting the results of cross-validation of the aforementioned equations, since in most cases, the results were satisfactory in group analyzes when verified by ANOVA, linear correlation coefficient, and simple linear regression but not at the individual level through the agreement provided by the analysis of Bland and Altman in 1986, and verification of measurement bias and trend. Thus, the researcher should choose the most appropriate equation according to their goal; group or individual analysis.

### Run/walk test over distances of 550 meters to 1 mile 

Among the protocols that consider pre-established fixed distances, the run/walk test of 1 mile is the most widespread and investigated in the literature, being used in a total of 5 papers that met the inclusion criteria and eligibility of this systematic review. ^16^ ^,^ ^27^ ^,^ ^28^ ^,^ ^33^ ^,^ ^37^

Validation research initiatives have used the equation of Cureton et al.^33^ to estimate VO_2_ peak in the 1-mile test and this fact can be justified by certain factors, such as it uses variables that are easy to access, and presents less intra and inter appraiser errors in the regression model (total time in the test, gender, age, and BMI), compared with the equation of Buono et al.^27^ ^,^ who use the measurement of skinfold thickness in their model, and is recommended by the FITNESSGRAM battery of tests, to calculate VO_2_peak when performing the 1-mile test to verify the CRF in young people. 

In relation to tests with pre-established distances, there was moderate evidence for the 1-mile run/walk test, and limited evidence for the 550 meters running, 1 mile walking, 1 mile submaximal, and 0.5 mile run/walk protocols, due to the lack of studies that aimed to validate these tests in young people. The equation proposed by Cureton et al.^33^ seems to be the best equation for estimating VO_2_peak for the 1 mile run/walk test, considering that the level of physical fitness of the individuals may influence the test results. Thus, our results are in agreement with those of Castro-Piñero et al.^8^ , since new initiatives for validating these test protocols were not identified in the literature.

### Run/walk tests for times of 6, 9, and 12 minutes

In the 6-minute walk test, there was limited evidence of validity with inconsistent results in the studies, which was also demonstrated by the 6-minute running test, with only one study that tested its validity. ^24^ This fact can be explained in part by the characteristics of the test, such as the duration and type of effort. On the other hand, the 9-minute test presented evidence of validity considered moderate. Four studies investigated the validity, and favorable results were obtained (r=0.43 to 0.71). ^22^ ^,^ ^32^ ^,^ ^39^ ^,^ ^49^ ^.^ Only one initiative to propose and validate an equation to estimate VO_2_peak in the 9-minute test was found, but despite the high quality of the paper, classification of the evidence was not possible due to its representation in only one paper. ^22^

As well as the running and walking 6-minute tests, the 12-minute run/walk test also demonstrated limited evidence of validity in young people, represented by only two moderate quality papers that verified the validity, ^39^ ^,^ ^53^ with only one equation proposed to estimate VO_2_ peak from the 12-minute test. ^53^ Therefore, we suggest future initiatives to verify the validity of the field protocols of running and/or walking for 6 and 12 minutes, in order to provide more consistent results in the population of children and adolescents.

### Maximal and submaximal bench tests and other protocols 

Four studies were found which assessed the validity of the bench test; 3 being maximal^27^ ^,^ ^38^ ^,^ ^41^ and one submaximal,^46^ with similar and favorable results (r=0.48 to r=0.84). Two proposed equations to estimate VO_2_ peak from the bench tests were presented, ^38^ ^,^ ^46^ with results considered valid for estimation of CRF, however, more cross-validation initiatives are still needed for evidence of its use in different populations. Thus, there is moderate evidence of validity for the bench test, but it is noteworthy that the tests featured differences, according to the protocol used.

The test protocol proposed by Andersen et al.^44^ was investigated in three papers, two of moderate quality^44^ ^,^ ^48^ and one of high quality.^21^ Thus, it was rated moderate evidence of validity, with no indication for the equation to estimate VO_2_ peak through the test, due to the limited number of papers that verified the validity of the original equation and the new proposal. ^21^ ^,^ ^44^ Furthermore, caution should be exercised when using the test of Andersen et al.,^44^ since it was designed for a young sample between 9 and 11 years old, and therefore, even with moderate evidence of validity, needs to be tested on samples of other ages before use.

## CONCLUSIONS

Given the results found in this systematic review, we conclude that the SR-20-m test seems to be the most appropriate to evaluate the CRF of young people, presenting strong evidence of validity. The equation recommended for estimation of VO_2_ peak from the SR-20-m test is that proposal by Barnett et al.^31^ with strong evidence and validity and, as an alternative, the proposals by Mahar et al.^43^ and Mahar et al.^47^ due to moderate evidence of validity, and being recommended by the FITNESSGRAM battery to estimate VO_2_ peak.

As a possible alternative for the evaluation of CRF, when using the SR-20-m test is impossible, the 1-mile test is indicated, which demonstrated moderate evidence of validity, as well as the equation proposed by Cureton et al.^33^ to estimate VO_2_peak from the 1-mile test. In addition, the 9-minute, bench and Andersen et al.^44^ tests can be used, which presented moderate evidence of validity; however, to date, there are no indication of equations to estimate VO_2_ peak through these tests.
